# Association of asthma with low serum vitamin D and its related musculoskeletal and psychological symptoms in adults: a case-control study

**DOI:** 10.1038/s41533-021-00239-7

**Published:** 2021-05-14

**Authors:** Mohammad J. Alkhatatbeh, Haneen S. Almomani, Khalid K. Abdul-Razzak, Shaher Samrah

**Affiliations:** 1 Department of Clinical Pharmacy, Faculty of Pharmacy, Jordan University of Science and Technology, Irbid, Jordan; 2 Department of Internal Medicine, Faculty of Medicine, Jordan University of Science and Technology, Irbid, Jordan

**Keywords:** Asthma, Epidemiology

## Abstract

There are complex potential inter-relationships between the chronic inflammation of asthma and poor control, vitamin D deficiency, musculoskeletal pain and anxiety and depression. The aim was to investigate associations between vitamin D and these possible co-morbidities. This case-controlled study involved 75 adults with asthma and 75 controls. Serum 25-hydroxyvitamin D (25(OH)D) was measured, levels of anxiety, depression, musculoskeletal pain, and asthma control were assessed. Participants with asthma had lower 25(OH)D and higher anxiety scores and higher measures of musculoskeletal pain compared to controls. Binary logistic regression showed that asthma was associated with decreased 25(OH)D (Odds ratio (OR) = 0.86), general weakness (OR = 13.29), complaint of musculoskeletal pain (OR = 13.73), and increased intensity of musculoskeletal pain (OR = 0.61) and number of painful sites (OR = 2.58). Asthma was not associated with anxiety or depression. Further studies are required to investigate if vitamin D supplementation can improve asthma symptoms and musculoskeletal pain.

## Introduction

Asthma is a chronic inflammatory airway disease that is characterized by bronchial hyper-responsiveness and tissue remodeling^1^. Asthma is usually triggered by a combination of genetic and environmental factors that end with inappropriate immune-mediated chronic inflammatory responses, which may influence the severity of the disease and response to treatment^1,2^. Therefore, patients with asthma may experience recurrent episodes of wheezing, coughing, shortness of breath, and chest tightness that interfere with physical activity, sleeping habits, and ultimately, quality of life^3^.

There is a possible association between asthma and certain psychological conditions including stress, anxiety, and depression^4^. This connection could be explained by an interaction between behavioral, neural, endocrine, and immune processes, which may trigger bronchial inflammatory responses, hyper-responsiveness and the development of asthma^4^. In addition, studies have reported that patients with asthma have higher prevalence of anxiety disorders, suggesting that the relationship between asthma and anxiety could be bidirectional^5,6^. Regardless of the nature of association between asthma and the psychological symptoms, these mental health issues can interfere with the optimal management of asthma and inversely affect asthma control^7^.

Interestingly, there is increased evidence suggesting an association between anxiety, depression, and certain nutrient deficiencies including vitamin D and calcium^8,9^. Vitamin D is involved in the function of the central nervous system (CNS) as its receptors are widely expressed in the human brain^10,11^. Consequently, low vitamin D levels have been found to be associated with anxiety and depression in patients with chronic pain such as fibromyalgia, musculoskeletal pain, and non-cardiac chest pain^12–14^. Low calcium levels, which could result from vitamin D deficiency^15^, could also have stimulatory effects on neuromuscular junctions, which may lead to certain psychological disorders including anxiety and depression^16^.

Vitamin D may also play a role in the pathogenesis of asthma^17^. Several studies have identified a link between vitamin D deficiency and both worsening of asthma symptoms and increased risk of asthma exacerbations in children and adults^17–20^. Asthmatic patients with low vitamin D levels have more severe symptoms and poor asthma control^21^. In addition, the severity of vitamin D deficiency correlates with the number of medications being used to control asthma, suggesting a possible link with treatment resistance^22^. The role of vitamin D in asthma has been proposed to be due to its immunomodulatory effect on the inflammatory responses of the airways^23^. Studies in vitro and in vivo have demonstrated that treatment with vitamin D is able to reduce inflammatory signaling in many cell types that are involved in the pathogenesis of asthma; inhibiting differentiation, maturation, and cytokine release from mast cells, neutrophils and eosinophils and effectively reducing airway hyper-responsiveness, inflammation, and remodeling^24^.

Low serum vitamin D has also been found to be associated with increased risk of musculoskeletal pain^14,25^. Individuals who experience musculoskeletal pain are more likely to have anxiety and depression^14^, which in turn could be associated with the development of asthma or worsening of its symptoms.

Therefore, the purpose of this study was to investigate the prevalence of vitamin D deficiency, musculoskeletal pain, anxiety, and depression among adult patients with asthma compared to healthy controls. In addition, we aimed to investigate the association of both asthma and level of its control with serum vitamin D levels, measures of musculoskeletal pain, anxiety, and depression. We hypothesized that low serum vitamin D, increased anxiety and depression scores, and increased measures of musculoskeletal pain are associated with asthma and poor asthma control.

## Results

### Characteristics of study participants

This study involved a total of 150 participants (46 males and 104 females): 75 participants with asthma and 75 age and gender-matched healthy controls. The mean (±standard deviation, SD) age of participants was 40.43 ± 12.81 years and the mean BMI was 28.57 ± 6.66 kg/m^2^. The mean HADS-anxiety and depression scores were 7.98 ± 4.53 and 6.68 ± 3.55, respectively. 72% (*n* = 108) of participants reported complaints of musculoskeletal pain in any site of the body with a median (25th–75th percentiles) number of painful body sites of 1 (1–3). The median serum 25(OH)D concentration was 12.96 (9.32–19.07) ng/mL, with 77.3% (*n* = 116) of participants classified as having deficient and 20% (*n* = 30) with insufficient vitamin D levels. Other socio-demographic characteristics of participants are shown in Table 1.

**Table 1:** General characteristics and differences in study variables between participants with asthma and healthy controls.

Variable	Total participants (*n* = 150)	Participants with asthma (*n* = 75)	Healthy controls (*n* = 75)	*P* value^*^
Age (Years)	40.43 ± 12.81	39.84 ± 13.67	41.01 ± 11.95	0.58
Gender				
Males	46 (30.7)	23 (30.7)	23 (30.7)	1.00
Females	104 (69.3)	52 (69.3)	52 (69.3)	
BMI (Kg/m^2^)	28.57 ± 6.66	28.51 ± 7.46	28.63 ± 5.80	0.92
Smoking				
Yes	26 (17.3)	8 (10.7)	18 (24.0)	0.03
No	124 (82.7)	67 (89.3)	57 (76.0)	
Marital status				
Single	41 (27.3)	26 (34.7)	15 (20.0)	0.04
Married	109 (72.7)	49 (65.3)	60 (80.0)	
Academic level				
Primary school	37 (24.7)	16 (21.3)	21 (28.0)	0.61
Secondary school	34 (22.7)	17 (22.7)	17 (22.7)	
High education	79 (52.7)	42 (56.0)	37 (49.3)	
Employment				
Yes	62 (41.3)	31 (41.3)	31 (41.3)	1.00
No (or retired)	88 (58.7)	44 (58.7)	44 (58.7)	
Number of family members	6.11 ± 2.25	6.24 ± 2.30	5.99 ± 2.21	0.49
Average family income (JD)				
≤500	94 (62.7)	46 (61.3)	48 (64.0)	0.94
501–1000	37 (24.7)	19 (25.3)	18 (24.0)	
>1000	19 (12.7)	10 (13.3)	9 (12.0)	
General weakness				
Yes	74 (49.3)	58 (77.3)	16 (21.3)	<0.001
No	76 (50.7)	17 (22.7)	59 (78.7)	
HADS-anxiety score (0–21)	7.98 ± 4.53	8.81 ± 4.38	7.15 ± 4.56	0.02
HADS-anxiety score (0–21)				
Normal (0–7)	73 (48.7)	31 (41.3)	42 (56.0)	0.08
Borderline (8–10)	26 (17.3)	12 (16.0)	14 (18.7)	
Abnormal (11–21)	51 (34.0)	32 (42.7)	19 (25.3)	
HADS-depression score (0–21)	6.68 ± 3.55	6.85 ± 3.63	6.51 ± 3.49	0.55
HADS-depression score (0–21)				
Normal (0–7)	88 (58.7)	43 (57.3)	45 (60.0)	0.22
Borderline (8–10)	41 (27.3)	18 (24.0)	23 (30.7)	
Abnormal (11–21)	21 (14.0)	14 (18.7)	7 (9.3)	
Complaint of musculoskeletal pain in any site of the body				
Yes	108 (72.0)	67 (89.3)	41 (54.7)	<0.001
No	42 (28.0)	8 (10.7)	34 (45.3)	
Intensity of musculoskeletal pain during the previous month (0–10)	0 (1–5)	5 (4–7)	3 (0–6)	<0.001
Number of painful body sites (1–12)	1 (1–3)	3 (1–5)	0 (0–1)	<0.001
25(OH)D (ng/mL)	12.96 (9.32–19.07)	12.31 (9.56–15.97)	15.50 (9.24–21.87)	0.04
25(OH)D (ng/mL)				
Deficient (<20 ng/mL)	116 (77.3)	67 (90.5)	49 (65.3)	<0.001
Insufficient (20–30 ng/mL)	30 (20.0)	7 (9.5)	23 (30.7)	
Sufficient (≥30 ng/ml)	3 (2.0)	0 (0)	3 (4.0)	

*BMI* Body Mass Index, *JD* Jordanian Dinar, *HADS* Hospital Anxiety and Depression Scale, *25(OH)D* 25-hydroxyvitamin D.

^*^Student’s *t*-test, Mann–Whitney’s *U* test, Chi-square test, or Fisher’s exact test as appropriate. *P* values < 0.05 were considered statistically significant. Data are presented as frequency (%), mean ± standard deviation, or median (25th–75th percentiles) as appropriate.

### Differences in study variables between participants with asthma and healthy controls

As shown in Table 1, participants with asthma had significantly lower overall levels of 25(OH)D (*p* value = 0.04), and were more likely to have deficient vitamin D levels compared to healthy controls (90.5% vs. 65.3%, *p* value <0.001). In addition, significantly more participants with asthma reported general weakness (77.3% vs. 21.3%, *p* value <0.001), musculoskeletal pain (89.3% vs. 54.7%, *p* value <0.001), higher intensity of musculoskeletal pain and significantly more painful body sites (*p* values <0.001) when compared to healthy controls, respectively. In addition, participants with asthma had significantly higher levels of HADS-anxiety scores compared to healthy controls (*p* value = 0.02). Comparison of other variables between participants with asthma and healthy controls are shown in Table 1.

### Association between asthma and serum 25(OH)D concentrations, HADS-anxiety scores, and measures of musculoskeletal pain

Binary logistic regression analysis (Table 2, dependent variable: asthma vs. healthy controls) revealed that asthma was significantly associated with increases in general weakness (OR = 13.29, *p* value <0.001), complaints of musculoskeletal pain (OR = 13.73, *p* value = 0.02), and number of painful body sites (OR = 2.58, *p* value <0.001), but decreases in intensity of musculoskeletal pain during the previous month (OR = 0.49, *p* value = 0.01), and serum 25(OH)D concentrations (OR = 0.86, *p* value <0.01). In contrast, asthma was not significantly associated with HADS-anxiety scores (OR = 1.00, *p* value = 0.98), HADS-depression scores (OR = 0.87, *p* value = 0.09), or smoking (OR = 3.74, *p* value = 0.05).

**Table 2:** Association between asthma and study variables.

Variable	Value	OR	Confidence interval	*P* value^a^
Constant	–	–	–	0.40
Smoking	Yes No (Reference)	3.74	0.98–14.28	0.05
General weakness	Yes No (Reference)	13.29	3.61–48.90	<0.001
Complaint of musculoskeletal pain in any site of the body	Yes No (Reference)	13.73	1.57–120.07	0.02
Intensity of musculoskeletal pain during the previous month	–	0.61	0.42–0.89	0.01
Number of painful body sites	–	2.58	1.57–4.21	<0.001
HADS-anxiety score	–	1.00	0.86–1.17	0.98
HADS-depression score	–	0.87	0.74–1.02	0.09
25(OH)D (ng/mL)	–	0.86	0.79–0.94	<0.01

*HADS* Hospital Anxiety and Depression Scale, *OR* Odds ratio, *25(OH)D* 25-hydroxyvitamin D.

^a^Binary logistic regression (dependent variable: asthma vs. controls), *p* values < 0.05 were considered statistically significant.

### Asthma-related characteristics among participants with asthma

The median duration of asthma among participants (*n* = 75) was 5 (2–14) years and the median ACT score was 17 (12–21). Accordingly, 33.3% (*n* = 25) of participants had well-controlled disease (ACT score > 20), compared to the 66.3% (*n* = 50) of participants with uncontrolled asthma (ACT score ≤ 20). The median number of medications used for the treatment of asthma including, inhaled corticosteroid, was 2 (1–2). The proportions of participants using low, medium, and high doses inhaled corticosteroids were: 41.3% (*n* = 31), 53.3% (*n* = 40), and 5.3% (*n* = 4), respectively. Participants reported a median number of 1 (0–3) emergency department visits due to asthma during the previous year.

### Differences in study variables between participants with uncontrolled and well-controlled asthma

As shown in Table 3, participants with uncontrolled asthma had significantly higher intensity of musculoskeletal pain during the previous month (*p* value = 0.04), higher number of painful body sites (*p* value = 0.01), higher number of medications used for asthma (*p* value = 0.01), and higher number of emergency department visits because of asthma during the previous year (*p* value <0.01) compared to participants with well-controlled asthma. Significantly more participants with well-controlled asthma were on low doses of inhaled corticosteroid compared to participants with uncontrolled asthma (64.0% vs. 30%, *p* value = 0.01). In contrast, there was no significant difference in age, gender, general weakness, HADS-anxiety and depression scores, complaint of musculoskeletal pain, serum 25(OH)D, duration of asthma, or other variables as listed in Table 3.

**Table 3:** Differences in study variables between participants with uncontrolled and well-controlled asthma.

Variable	Participants with uncontrolled asthma (ACT score ≤ 20) *n* = 50	Participants with well-controlled asthma (ACT score = 20–25) *n* = 25	*P* value^*^
Age (Years)	38.88 ± 13.37	41.76 ± 14.35	0.39
Gender			
Males	18 (36.0)	5 (20.0)	0.16
Females	32 (64.0)	20 (80.0)	
BMI (Kg/m^2^)	28.40 ± 8.56	28.75 ± 4.70	0.85
Smoking			
Yes	44 (88.0)	23 (92.0)	0.71
No	6 (12.0)	2 (8.0)	
Marital status			
Single	18 (36.0)	8 (32.0)	0.73
Married	32 (64.0)	17 (68.0)	
Academic level			
Primary school	12 (24.0)	4 (16.0)	0.59
Secondary school	12 (24.0)	5 (20.0)	
High education	26 (52.0)	16 (64.0)	
Employment			
Yes	23 (46.0)	8 (32.0)	0.25
No (or retired)	27 (54.0)	17 (68.0)	
Number of family members	6.26 ± 2.63	6.20 ± 1.44	0.92
Average family income (JD)			
≤500	29 (58.0)	17 (68.0)	0.78
501–1000	14 (28.0)	5 (20.0)	
>1000	7 (14.0)	3 (12.0)	
General weakness			
Yes	41 (82.0)	17 (68.0)	0.17
No	9 (18)	8 (32.0)	
HADS-anxiety score (0–21)	9.16 ± 4.08	8.12 ± 4.94	0.34
HADS-anxiety score (0–21)			
Normal (0–7)	20 (40.0)	11 (44.0)	0.47
Borderline (8–10)	10 (20.0)	2 (8.0)	
Abnormal (11–21)	20 (40.0)	12 (48.0)	
HADS-depression score (0–21)	7.08 ± 3.50	6.40 ± 3.92	0.45
HADS-depression score (0–21)			
Normal (0–7)	28 (56.0)	15 (60.0)	0.89
Borderline (8–10)	13 (26.0)	5 (20.0)	
Abnormal (11–21)	9 (18.0)	5 (20.0)	
Complaint of musculoskeletal pain in any site of the body			
Yes	45 (90.0)	22 (88.0)	0.79
No	5 (10.0)	3 (12.0)	
Intensity of musculoskeletal pain during the previous month (0–10)	6 (4–7.5)	5 (3–6)	0.04
Number of painful body sites (1–12)	4 (1–5.25)	1 (0–3)	0.01
25(OH)D (ng/mL)	12.91 (10–16.42)	11.59 (7.87–15.36)	0.30
Vitamin D status			
Deficient (<20 ng/mL)	45 (91.8)	22 (88.0)	0.68
Insufficient (20–30 ng/mL)	4 (8.2)	3 (12.0)	
Sufficient (≥30 ng/ml)	0 (0)	0 (0)	
Duration of asthma (Years)	5 (2–11.25)	7 (3–19.5)	0.30
Number of medications used for asthma	2 (2–2)	2 (1–2)	0.01
Number of emergency department visits because of asthma during the previous year	2 (0–5)	0 (0–1)	<0.01
Inhaled corticosteroid dose			
Low dose	15 (30.0)	16 (64.0)	0.01
Medium dose	31 (62.0)	9 (36.0)	
High dose	4 (8.0)	0 (0)	
ACT score	12.94 ± 4.16	21.88 ± 1.51	<0.001

*ACT* Asthma Control Test, *BMI* Body Mass Index, *JD* Jordanian Dinar, *HADS* Hospital Anxiety and Depression Scale, *25(OH)D* 25-hydroxyvitamin D.

^*^Student’s *t*-test, Mann–Whitney’s *U* test, Chi-square test, or Fisher’s exact test as appropriate. *P* values < 0.05 were considered statistically significant. Data are presented as frequency (%), mean ± standard deviation, or median (25th–75th percentiles) as appropriate.

### Association between levels of asthma control and other study variables

As shown in Table 4, uncontrolled asthma was significantly associated with younger age (OR = 0.92, *p* value = 0.01), male gender (OR = 0.14, *p* value = 0.04), and increased number of painful body sites (OR = 1.58, *p* value = 0.04). In contrast, there was no significant association between the level of asthma control and BMI (OR = 0.98, *p* value = 0.74), smoking (OR = 0.22, *p* value = 0.25), general weakness (OR = 2.16, *p* value = 0.35), complaint of musculoskeletal pain (OR = 0.09, *p* value = 0.09), intensity of musculoskeletal pain (OR = 1.58, *p* value = 0.07), HADS-depression scores (OR = 1.05, *p* value = 0.65), or serum 25(OH)D (OR = 1.05, *p* value = 0. 59).

**Table 4:** Association between level of asthma control and other study variables.

Variable	Value	OR	Confidence interval	*P* value^*^
Constant	-	-	-	0.11
Age (Year)	-	0.92	0.86-0.98	0.01
Gender	Female Male (Reference)	0.14	0.02-0.91	0.04
BMI (kg/m^2^)	-	0.98	0.86–1.11	0.74
Smoking	Yes No (Reference)	0.22	0.02–2.91	0.25
Academic level	Primary school Secondary school High education (Reference)	1.80 0.52	0.30–10.62 0.11–2.58	0.52 0.43
General weakness	Yes No (Reference)	2.16	0.43–10.72	0.35
Complaint of musculoskeletal pain in any site of the body	Yes No (Reference)	0.09	0.01–1.47	0.09
Intensity of musculoskeletal pain during the previous month (0–10)	-	1.58	0.97–2.58	0.07
Number of painful body sites	-	1.58	1.03–2.32	0.04
HADS-anxiety score	-	1.01	0.84–1.21	0.93
HADS-depression score	-	1.05	0.86–1.28	0.65
25(OH)D (ng/mL)	-	1.05	0.89–1.23	0.59

*BMI* body mass index, *HADS* Hospital Anxiety and Depression Scale, *OR* Odds ratio, *25(OH)D* 25-hydroxyvitamin D.

^*^Binary logistic regression (dependent variable: uncontrolled asthma vs. controlled asthma), *p* values < 0.05 were considered statistically significant.

## Discussion

The prevalence of vitamin D deficiency and insufficiency among both participants with asthma and healthy controls was very high (77.3% and 20.0%, respectively) in the recruited cohort. A major finding was that participants with asthma had significantly lower levels of serum 25(OH)D compared to healthy controls, and were thus also more likely to be classified as being vitamin D deficient compared to healthy controls (90.5% vs. 65.3%, respectively). Consistent with our hypothesis, participants with asthma also had significantly higher measures of vitamin D deficiency symptoms including general weakness, musculoskeletal pain, and anxiety. Hence, the current study has shown a significant association between asthma and low levels of serum 25(OH)D and its related musculoskeletal symptoms (Table 2) but it has not shown any significant association between lower levels of 25(OH)D and levels of asthma control, as indicated by the ACT scores (Table 4). Although anxiety was more prevalent in participants with asthma compared to healthy controls, there was no significant association between asthma and HADS-anxiety scores. Also, there was no significant difference in HADS-depression scores between participants with asthma and healthy controls and there was no significant association of asthma with HADS-depression scores. HADS-anxiety and depression scores were also not significantly different between participants with uncontrolled asthma and participants with well-controlled asthma. Moreover, there was no significant association between HADS-anxiety and depression scores and the level of asthma control.

Our results build on previous findings by Samrah et al.^26^ who showed that serum 25(OH)D was significantly lower in Jordanian women with asthma compared to healthy controls. This is despite the different methods used to assess serum 25(OH)D, whereby Samrah et al.^26^ employed liquid chromatography-tandem mass spectrometry (LC-MS/MS) compared to the electrochemiluminescence immunoassay that was used in the current study. The high prevalence of low vitamin D levels among participants with asthma is also consistent with Solidoro et al.^19^, in which low serum vitamin D was reported in 111 out of 119 participants with asthma, and confirms other studies reporting lower levels of serum 25(OH)D in participants with asthma compared to healthy controls^27–30^. These results may suggest a role for vitamin D in asthma, which is supported by the significant immunomodulatory effect of vitamin D in both innate and adaptive immunity^31–33^. This role could also be supported by the significant association between asthma and low levels of serum 25(OH)D and its related musculoskeletal symptoms that was revealed by the regression analysis in Table 2. On the contrary, other studies showed opposite findings, demonstrating that low 25(OH)D was not significantly associated with asthma^34–36^. This inconsistency could be explained by the difference in study populations, as the prevalence of both asthma and vitamin D deficiency may be affected by ethnicity^37,38^.

In recent years, several clinical studies have concluded that vitamin D is deficient in global populations; it is a worldwide health problem affecting more than one billion children and adults^39,40^. A recent study showed that 89.7% of Jordanian adults had vitamin D insufficiency and deficiency (25(OH)D < 30 ng/ml), especially women^41^. Lifestyle and behavioral changes like spending more time indoors, avoiding sunlight, and the increasing use of sunblock may be the major contributors to this vitamin deficiency epidemic^42,43^.

Although asthma is a heterogeneous disorder and is influenced by many immunological factors, growing evidence has linked vitamin D with asthma pathogenesis^44^. This connection may be explained by the presence of abundant vitamin D receptors in airway tissue as well as the known existence of vitamin D receptor polymorphisms^45,46^. Vitamin D may have anti-inflammatory effects as it can inhibit the production of pro-inflammatory cell factors and promote T-regulatory cell secretion of anti-inflammatory cytokines^45,46^. Because vitamin D deficiency was reported to be associated with higher blood eosinophil counts^47^, a randomized controlled trial of vitamin D supplementation was conducted and shown to decrease eosinophilic airway inflammation in patients with non-atopic asthma with severe eosinophilic bronchial inflammation^48^.

The lack of association between lower levels of serum 25(OH)D and asthma control is in parallel with the Norwegian study of asthmatic adults (HUNT study)^49^, and another study conducted in the UK^50^. Observational studies have consistently indicated a positive correlation between lower levels of vitamin D and severity of asthma mostly in children^51–53^ but fewer in adults^21,54^. Although serum 25(OH)D was not significantly different between participants with uncontrolled asthma and those with controlled asthma in the current study, measures of musculoskeletal pain, which is a symptom of vitamin D deficiency, were significantly higher in participants with uncontrolled asthma compared to participants with controlled asthma. In addition, uncontrolled asthma was significantly associated with an increased number of painful body sites (Table 4).

The prevalence of abnormal anxiety scores reported in our asthmatic participants (42.7%) was similar to the prevalence reported by Baumeister et al.^55^. Our results regarding the lack of association between anxiety and asthma control was consistent with Trzcinska et al.^56^, but the lack of association between depression and asthma control was inconsistent with the same study^56^, in which depression was significantly correlated with the degree of asthma control.

The major strength of our study was its case-control design that involved a comparison between participants with asthma and age/gender matched healthy controls. Also, results of this study are supported by the well-validated methods that were used to assess different variables including serum 25(OH)D levels, anxiety, depression, musculoskeletal pain, and level of asthma control. In contrast, the current study had some limitations that could prevent the conclusions from being inferred to the general population. For instance, data about asthma control, anxiety, and depression were assessed by self-reporting. However, the questionnaires that were used to assess these conditions were well-validated, reliable, widely used, and approved for clinical research purposes. In addition, proper inhalation techniques and adherence to asthma medications were not investigated as we relied on subjective physician/pharmacist assessment. Furthermore, we should also acknowledge that by only including participants with (self-reported) adherence, we are limiting the generalizability of the findings to the minority of people with asthma who are adherent. Moreover, we were unable to recruit asthmatic patients and controls with sufficient vitamin D levels as vitamin D deficiency was highly prevalent in the study population. So, we were not able to compare study variables with participants with sufficient vitamin D levels. Regardless of these limitations, we still believe that the results of the present study are valid and contribute important information to the literature. Our results may encourage other researchers to investigate if there is a causal relationship between vitamin D deficiency and asthma. Also, further studies are required to investigate if vitamin D supplementation may improve asthma symptoms, general weakness, and musculoskeletal pain in patients with asthma.

In conclusion, vitamin D deficiency was highly prevalent in the both participants with asthma and healthy controls, but serum levels of 25(OH)D among participants with asthma were significantly lower compared to healthy controls. In addition, vitamin D deficiency symptoms including general weakness, musculoskeletal pain, and anxiety were highly prevalent among participants with asthma compared to healthy controls. Asthma was significantly associated with decreased serum 25(OH)D levels, and complaints of general weakness and musculoskeletal pain. In contrast, there was no significant association between asthma and either anxiety or depression. Measures of musculoskeletal pain were significantly higher in participants with uncontrolled asthma compared to participants with controlled asthma. There was no association between the degree of asthma control and serum 25(OH)D, anxiety, or depression. However, uncontrolled asthma was significantly associated with younger age, male gender, and increased number of painful body sites.

## Methods

### Study design and participants

This is a case-control study that involved 75 participants with asthma and 75 age and gender-matched healthy controls (age ranged from 16 to 66 years). Sample size was determined based on the mean ± SD of 25(OH)D for healthy adults (17.35 ± 9.81 ng/mL) as reported in our previous study^13^. Based on this standard deviation, we calculated that a minimum sample size of 50 was sufficient to detect a mean difference of ±6.5 between the groups with 90% power, which was considered clinically relevant. The study was conducted between October 2019 and July 2020. Participants with asthma were recruited from the adults’ pulmonary clinic at King Abdullah University Hospital (KAUH), Irbid, Jordan. Healthy controls were recruited from healthy individuals who visited KAUH for other purposes. All asthmatic patients who visited the clinic during the study period were eligible for the study. Asthma diagnosis was dependent on the presence of asthma symptoms and confirmed by a consultant pulmonologist using a diagnostic spirometry. The presence of asthma was defined by having at least one attack with typical asthma symptoms including cough, chest tightness, dyspnea, and wheezes during the previous year^22^. Asthmatic patients with any of the following conditions were excluded from the study: patients who were not adherent to their asthma medications during the previous three months or not using these medications properly as judged by the pharmacist or the consultant pulmonologist and based on patients’ self-reporting, patients with a history of vitamin D supplementation during the previous 3 months, patients with medical conditions that may affect the level of 25(OH)D including, chronic kidney disease, chronic liver disease, malabsorption, inflammatory disease like rheumatoid arthritis, and systemic lupus erythematosus, or women with pregnancy or lactation, patients with conditions that affect the degree of asthma control (such as upper and lower respiratory tract infections, bronchitis, or emphysema), patients with cancer, or patients who only had asthma symptoms during exercise and never at other times. We only included participants with good adherence to asthma medications to exclude poor adherence as a possible cause for uncontrolled asthma and thus the relationship between study variables. The study protocol was approved by the Institutional Review Board of Jordan University of Science and Technology. Every participant provided a signed consent form and was informed about the research goals and details.

### Data collection

Self-reported data collection included: age, gender, smoking, marital status, height (cm), weight (kg), academic level, employment, number of family members, average family income, feeling of general weakness, and number of emergency department visits because of asthma during the previous year. Body Mass Index (BMI) was calculated in kg/m^2^. Data about duration of asthma, number of medications used for asthma, and dose of inhaled corticosteroid medications were obtained from participants’ medical records. Inhaled corticosteroid doses were divided into three groups: low dose, moderate dose, and high dose, according to the Global Initiative for Asthma (GINA) guideline, 2020.

### Assessment of asthma control

The level of asthma control was assessed by self-reporting using an Arabic version of the well-validated Asthma Control Test (ACT)^57,58^. The ACT is a simple to administer and clinically valid test for asthma control evaluation^57,58^. The ACT consists of 5 items measuring each of the followings over the preceding 4 weeks: (1) the impact of asthma on everyday life, (2) the recurrence of shortness of breath, (3) frequency of nighttime awakening due to asthma symptoms, (4) frequency of using rescue inhaler or nebulizer medication, and (5) description of rate of overall asthma control^57,58^. Each item comprises 5 choices with a value ranging from 1 to 5. For scoring, the ACT answer’s values are summed up for each of the five items to give a score range from 5 (poorly controlled asthma) to 25 (completely controlled asthma)^57,58^. An ACT score >20 reflects well-controlled asthma and ≤20 reflects uncontrolled asthma^59^.

### Assessment of anxiety and depression

Anxiety and depression were assessed by self-reporting using an Arabic version^13^ of the well-validated Hospital Anxiety and Depression Scale (HADS)^60^. HADS consists of 14 questions, seven questions refer to symptoms of anxiety and the other seven questions refer to symptoms of depression. Each question is coded from 0 to 3. Thus, the HADS-anxiety and depression scores can range from 0 to 21. Three HADS cut-off points have been proposed: a score of 0–7 does not suggest the presence of anxiety or depression (normal); a score of 8–10 refers to the presence of symptoms of moderate anxiety or depression (borderline); and a score of 11–21 refers to the presence of significant anxiety or depression (abnormal)^61^.

### Assessment of musculoskeletal pain

Participants were asked to report their sites of chronic musculoskeletal pain. Areas of musculoskeletal pain were recorded from the following body sites: neck, shoulders, lower back, hands, wrists, palms, arms, upper legs, knees, lower legs, hips, and feet. The number of painful body sites was summed. In addition, participants were asked to report the average intensity of musculoskeletal pain during the previous month using a 0–10 numerical rating scale (0 reflects no musculoskeletal pain and 10 reflects the maximum intensity of musculoskeletal pain)^62^.

### Blood sampling and assessment of serum 25 (OH) D

Venous blood samples were collected by a well-trained lab technician into anticoagulant-free plain test tubes. Then, serum was prepared by centrifuging blood samples at 2100 g for 8 min at room temperature within 2 h of blood collection using a high-speed Jouan centrifuge (Thermo Fisher Scientific, Inc., Waltham, MA, USA). Serum 25 (OH) D levels were determined by electrochemiluminescence immunoassay using Roche Modular E170 Analyzer (Roche Diagnostics, Basel, Switzerland). Participants were classified according to their serum 25 (OH) D into sufficient vitamin D levels (≥30 ng/mL), insufficient vitamin D levels (20–30 ng/mL), or deficient vitamin D levels (<20 ng/mL)^63^.

### Statistical analyses

Analysis was performed using the IBM SPSS Statistics software version 23 (Armonk, New York, USA). Continuous variables were tested for normality of distribution before analysis. Data were presented as frequency (%), mean ± standard deviation or median (25th–75th percentiles) as appropriate. Differences in qualitative variables between participants with asthma and healthy controls and between participants with controlled asthma and participants with uncontrolled asthma were determined using Chi-squared test or Fisher’s exact test as appropriate. Differences in quantitative study variables between participants with asthma and healthy controls and between participants with controlled asthma and participants with uncontrolled asthma were determined using the Student’s t-test or Mann-Whitney’s U test as appropriate. Association between asthma and serum 25(OH)D concentrations, HADS-anxiety scores, and measures of musculoskeletal pain were determined using Binary logistic regression analysis (dependent variable: asthma vs. controls). Association between level of asthma control and other study variables was also determined by Binary logistic regression analysis (dependent variable: uncontrolled asthma vs. controlled asthma). All *p* values were two-tailed and considered statistically significant at <0.05.

### Reporting summary

Further information on research design is available in the Nature Research Reporting Summary linked to this article.

## Supplementary information

Reporting Summary

## Data Availability

The data sets generated during and/or analyzed during the current study are available from the corresponding author on reasonable request.
