# Supplementary material for: Association of asthma with low serum vitamin D and its related musculoskeletal and psychological symptoms in adults: a case-control study
Source: NPJ Prim Care Respir Med. 2021 May 14;31:27. doi: 10.1038/s41533-021-00239-7 (PMC8121852; doi:10.1038/s41533-021-00239-7)
Supplement: Supplementary file 1 — Reporting Summary [file 41533_2021_239_MOESM1_ESM.pdf]

nature research

Corresponding author(s): DBRP: NPJPCRM-01170R

Last updated by author(s): Apr 7, 2021

Reporting Summary

Nature Research wishes to improve the reproducibility of the work that we publish. This form provides structure for consistency and transparency in reporting. For further information on Nature Research policies, see our Editorial Policies and the Editorial Policy Checklist.

Statistics

For all statistical analyses, confirm that the following items are present in the figure legend, table legend, main text, or Methods section.

n/a	Confirmed	
☐	☒	The exact sample size (*n*) for each experimental group/condition, given as a discrete number and unit of measurement
☐	☒	A statement on whether measurements were taken from distinct samples or whether the same sample was measured repeatedly
☐	☒	The statistical test(s) used AND whether they are one- or two-sided<br>*Only common tests should be described solely by name; describe more complex techniques in the Methods section.*
☐	☒	A description of all covariates tested
☐	☒	A description of any assumptions or corrections, such as tests of normality and adjustment for multiple comparisons
☐	☒	A full description of the statistical parameters including central tendency (e.g. means) or other basic estimates (e.g. regression coefficient) AND variation (e.g. standard deviation) or associated estimates of uncertainty (e.g. confidence intervals)
☐	☒	For null hypothesis testing, the test statistic (e.g. *F*, *t*, *r*) with confidence intervals, effect sizes, degrees of freedom and *P* value noted<br>*Give P values as exact values whenever suitable.*
☒	☐	For Bayesian analysis, information on the choice of priors and Markov chain Monte Carlo settings
☒	☐	For hierarchical and complex designs, identification of the appropriate level for tests and full reporting of outcomes
☐	☒	Estimates of effect sizes (e.g. Cohen's *d*, Pearson's *r*), indicating how they were calculated

Our web collection on statistics for biologists contains articles on many of the points above.

Software and code

Policy information about availability of computer code

Data collection	IBM SPSS Statistics software version 23 (Armonk, New York, USA).
Data analysis	IBM SPSS Statistics software version 23 (Armonk, New York, USA).

For manuscripts utilizing custom algorithms or software that are central to the research but not yet described in published literature, software must be made available to editors and reviewers. We strongly encourage code deposition in a community repository (e.g. GitHub). See the Nature Research guidelines for submitting code & software for further information.

Data

Policy information about availability of data

All manuscripts must include a data availability statement. This statement should provide the following information, where applicable:

- Accession codes, unique identifiers, or web links for publicly available datasets
- A list of figures that have associated raw data
- A description of any restrictions on data availability

The data sets generated during and/or analysed during the current study are available from the corresponding author on reasonable request.

April 2020

Field-specific reporting

Please select the one below that is the best fit for your research. If you are not sure, read the appropriate sections before making your selection.

☒ Life sciences ☐ Behavioural & social sciences ☐ Ecological, evolutionary & environmental sciences

For a reference copy of the document with all sections, see nature.com/documents/nr-reporting-summary-flat.pdf

Life sciences study design

All studies must disclose on these points even when the disclosure is negative.

Sample size	Sample size was determined based on the mean ±SD of 25(OH)D for healthy adults (17.35± 9.81 ng/mL) as reported in our previous study. Based on this standard deviation, we calculated that a minimum sample size of 50 was sufficient to detect a mean difference of ±6.5 between the groups with 90% power, which was considered clinically relevant
Data exclusions	Asthmatic patients with any of the following conditions were excluded from the study: patients who were not completely adherent to their asthma medications during the previous three months or not using these medications properly as judged by the pharmacist or the consultant pulmonologist and based on patients' self-reporting, patients with a history of vitamin D supplementation during the previous 3 months, patients with medical conditions that may affect the level of 25(OH)D including, chronic kidney disease, chronic liver disease, malabsorption, inflammatory disease like rheumatoid arthritis, and systemic lupus erythematosus , or women with pregnancy or lactation, patients with conditions that affect the degree of asthma control (such as upper and lower respiratory tract infections, bronchitis, or emphysema), patients with cancer, or patients who only had asthma symptoms during exercise and never at other times.
Replication	No experiments was conducted. The study was case controlled that involved data collection and blood sampling to measure vitamin D concentration for all participants by electrochemiluminescence immunoassay in the hospital laboratory.
Randomization	The sample was convenient. Recruitment was described in detail in the Methods section.
Blinding	The study was case controlled that involved data collection and blood sampling to measure vitamin D concentration for all participants by electrochemiluminescence immunoassay in the hospital laboratory. There was no need for blinding.

Reporting for specific materials, systems and methods

We require information from authors about some types of materials, experimental systems and methods used in many studies. Here, indicate whether each material, system or method listed is relevant to your study. If you are not sure if a list item applies to your research, read the appropriate section before selecting a response.

Materials & experimental systems

n/a	Involved in the study	
☒	☐	Antibodies
☒	☐	Eukaryotic cell lines
☒	☐	Palaeontology and archaeology
☒	☐	Animals and other organisms
☐	☒	Human research participants
☒	☐	Clinical data
☒	☐	Dual use research of concern

Methods

n/a	Involved in the study	
☒	☐	ChIP-seq
☒	☐	Flow cytometry
☒	☐	MRI-based neuroimaging

Human research participants

Policy information about studies involving human research participants

Population characteristics	This is a case controled study that involved 75 participants with bronchial asthma and 75 age and gender matched healthy controls (age ranged from 16 to 66 years).
Recruitment	The study was conducted between October 2019 and July 2020. Participants with asthma were recruited from the adults' pulmonary clinic at King Abdullah University Hospital (KAUH), Irbid, Jordan. Healthy controls were recruited from healthy individuals who visited KAUH for other purposes. All asthmatic patients who visited the clinic during the study period were eligible for the study. Asthma diagnosis was dependent on the presence of asthma symptoms and confirmed by a consultant pulmonologist using a diagnostic spirometry. The presence of asthma was defined by at least one attack with typical asthma symptoms including cough, chest tightness, dyspnea, and wheezes during the previous year.
Ethics oversight	The study protocol was approved by the Institutional Review Board of Jordan University of Science and Technology. Every participant provided a signed consent form and was informed about the approval of the study protocol must also be provided in the manuscript.

Note that full information on the approval of the study protocol must also be provided in the manuscript.
